# Necked strawberries are especially susceptible to cracking

**DOI:** 10.7717/peerj.15402

**Published:** 2023-05-11

**Authors:** Grecia Hurtado, Moritz Knoche

**Affiliations:** Institute for Horticultural Production Systems, Leibniz University Hannover, Hannover, Germany

**Keywords:** *Fragaria × ananassa*, Splitting, Rupture, Microcrack, Macrocrack, Cuticle

## Abstract

Fruit cracking is a commercially important disorder that reduces both quantity and quality of strawberries (*Fragaria* × *ananassa* Duch.). The objective was to identify the physiological mechanism of cracking and the factors affecting cracking. Cracking is more common in necked than in normal-shaped fruit. Most macroscopic cracks (‘macrocracks’) occur in the seedless neck. Large fruit is more cracking susceptible than medium size or small fruit. Macrocrack orientation is predominantly latitudinal in the proximal region of the neck and longitudinal in the mid and distal regions of the neck. The neck region of necked fruit has a thicker cuticle than the body of necked or normal-shaped fruit. The vascular bundles in the neck (seedless) are orientated longitudinally, while those in the body (with seeds) are both longitudinal and radial. Epidermal cells in the neck region are elongated longitudinally, with those in the proximal region of the neck being more elongated than those in the mid or distal regions of the neck. Cuticular microcracking was more severe in necked fruit than in normal-shaped fruit. The orientations of the microcracks matched those of the macrocracks, *i.e*., latitudinal in the proximal neck and longitudinal in the mid and distal neck regions. Following artificial incisions (blade), gaping was significantly more pronounced in necked than in normal-shaped fruit. Incubation of fruit in deionized water induced macrocracks in about 75% of fruit. Necked fruit cracked more than normal-shaped fruit. Most macrocracks were oriented latitudinally in the proximal neck and longitudinally in the distal neck regions. The results indicate cracking results from excessive growth strains which are further increased by surface water uptake.

## Introduction

Strawberry fruit is highly perishable. Its quality is often compromised by pre- and postharvest disorders. Impaired fruit quality causes significant economic loss during harvesting, packing and marketing (Herrington et al., 2009).

Preharvest disorders arise from environmental factors during growth and development. Among these, rain damage is a critical factor in all regions where rain occurs during the fruiting season (Blanco, Santos & Romero, 2006; Herrington et al., 2009, 2011, 2013; Menzel, 2021). The high susceptibility of strawberry to rain damage has caused a shift in production from the open field to greenhouses or plastic tunnels—but these are more capital intensive (Menzel, Smith & Moisander, 2014).

Rain damage in strawberries includes water soaking and cracking (Herrington et al., 2009).

Water soaking is a surface disorder that occurs in unprotected field production when strawberries are exposed to rain. Symptomatic fruit show pale, deliquescent patches of skin (Herrington et al., 2009, 2013; Hurtado & Knoche, 2021). Recently, the physiological mechanism of water soaking has been elucidated and factors affecting water soaking have been identified (Hurtado & Knoche, 2021). To our knowledge there are no such studies on cracking in strawberries, and the physiological mechanism of cracking in strawberries is unknown.

Cracking is a failure of the fruit skin that can be categorized as both microcracking (invisible) and macrocracking (visible). Macrocracks are always preceded by the formation of microcracks. Microcracks are minute fractures whose depth is limited to the thickness of the cuticle (<1 µm). These cannot be observed by the naked eye. Macrocracks are easily observed. They extend through the skin into the flesh (~2–3 mm) (Knoche & Winkler, 2017). In strawberries, macrocracks have been observed in the shoulder and in the neck of the fruit (Herrington et al., 2011).

Understanding the physiological mechanism of cracking is a necessary prerequisite for developing effective mitigating strategies—either through changes in cultural practice or through breeding or both. For this reason, the objective of this study is to identify the mechanism of cracking and the factors affecting cracking in strawberry fruit.

## Materials and Methods

### Plant material

Strawberry fruits were harvested from a commercial plantation in the open field at Gleidingen (lat. 52°16′N, long. 9°50′E), and from the Horticultural Research Station in Ruthe (lat. 52°14′N, long. 9°49′E), and from a growth chamber at the Herrenhausen Campus (lat. 52°23′N, long. 9°42′E) of the Leibniz University of Hannover, Germany. Temperature and relative humidity (RH) of the growth chamber were set at 20/16 °C and 60/80% RH during a 16/8 h day/night photoperiod. Fruit of the following strawberry cultivars were used: ‘Clery’, ‘Faith’, ‘Dream’, ‘Joly’ and ‘Lambada’.

Unless otherwise specified, uniform and sound fruit were harvested randomly at commercial ripeness (>80% of the fruit surface red) (Mitcham, Crisosto & Kader, 1996). Fruit was processed fresh on the day of collection or after a maximum of 2 d of storage at 2 °C and 80% RH.

### Macroscopic analysis

**Figure 1:**
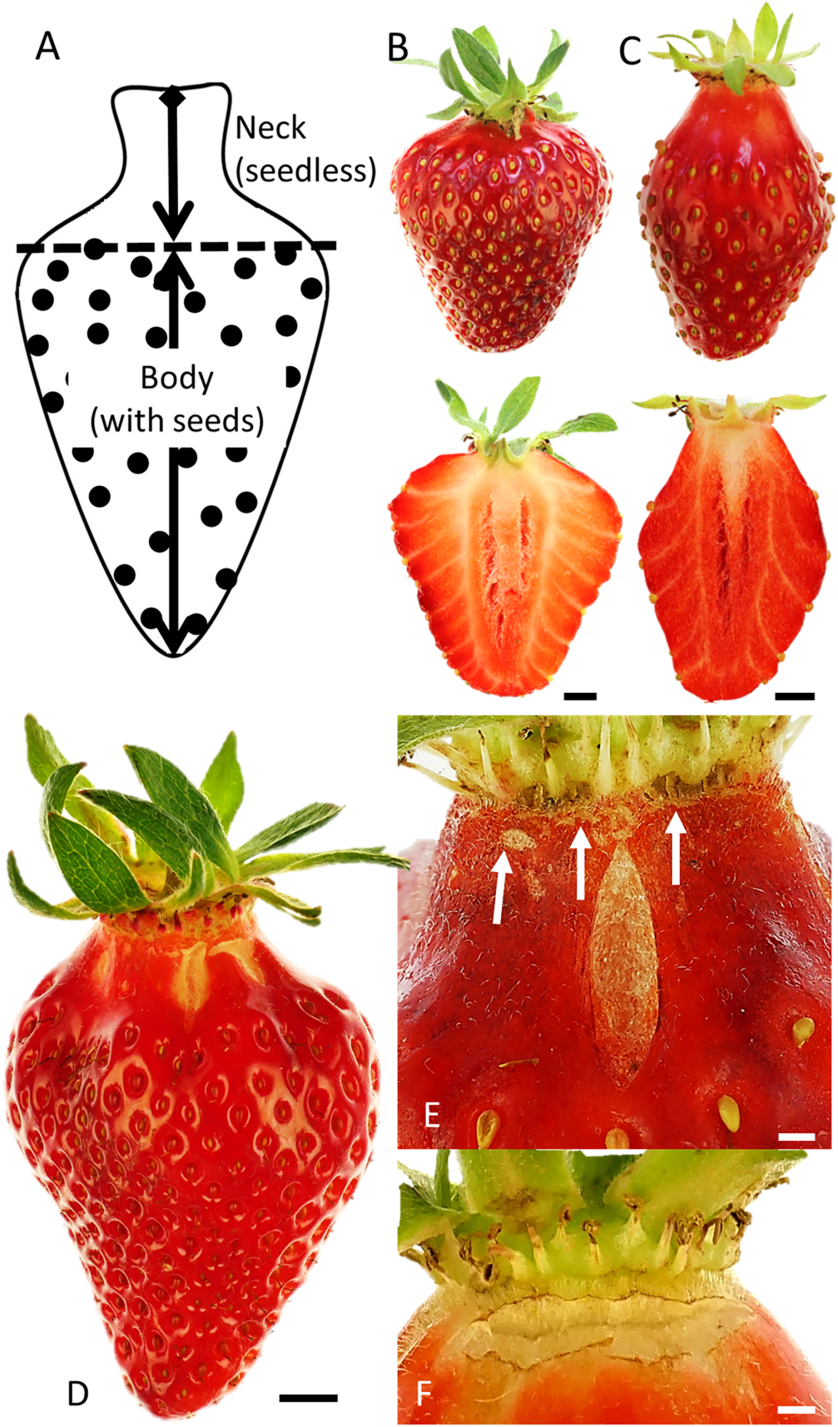
Strawberry fruit without and with neck and macroscopic cracks. (A) Sketch illustrating the nomenclature used to distinguish the neck (seedless) from the body (with seeds) of a mature strawberry fruit. For simplicity we refer to the strawberry pseudocarp as a ‘fruit’ and its achenes as ‘seeds’ even though *sensu stricto* they are not. Top view and longitudinal section of (B) normal-shaped or (C) necked strawberries. (D) Strawberry fruit with macrocracks. Detailed view of (E) a longitudinal macrocrack in the mid region of the seedless zone or ‘neck’ and (F) of a latitudinal macrocrack in the proximal region of the neck. White arrows in E indicate latitudinal cracks in the proximal region of the neck. Scale bars in B, C, D = 5 mm E, F = 1 mm.

The position and orientation of macrocracks on the fruit surface and the shape of the fruit were established on fruit from Gleidingen. Fruit shape was classified into two categories—‘normal’ and ‘necked’ fruit—based on the length of the seedless zone *i.e*., the so-called ‘fruit neck’ (Fig. 1A). Normal fruit were of conical shape with a very short ‘neck’ that was frequently covered by the calyx (Fig. 1B). In necked fruit, the conical shape was elongated, and the neck was more extended (Fig. 1C). Three fruit-size classes were inspected: small <11 g, medium size 11–22 g and large fruit >22 g. The experiment was conducted using ‘Faith’, ‘Dream’, ‘Joly’ and ‘Lambada’. The number of replicates was 50 per size class and cultivar.

Fruit were brought to the laboratory and calibrated photographs were taken (Lumix DMC-G80; Panasonic Corporation, Osaka, Japan) and the masses of cracked fruit from the field was determined. The length and width of the neck and of the body of each fruit and the orientations of any macrocracks relative to the fruit’s longitudinal axis were measured by image analysis (cellSens Dimension 1.7.1; Olympus Soft Imaging Solutions, Münster, Germany). This data was also used to establish objective criteria for categorizing fruit as necked *vs* normal shaped fruit.

### Cuticular mass

The mass per unit area of the cuticular membrane (CM) of the seedless neck and of the seeded body of both normal-shaped and necked fruit was determined. Epidermal discs comprising cuticle, epidermis, and some adhering flesh were excised using a biopsy punch (4 mm diameter; Kai Europe, Solingen, Germany). The CMs were enzymatically isolated by incubating discs in a solution of 50 mM citric acid buffer containing pectinase (90 ml l^−1^; Panzym Super E flüssig, Novozymes A/S, Krogshoejvej, Bagsvaerd, Denmark), cellulase (5 ml l^−1^; Cellubrix L; Novozymes A/S) and 30 mM NaN_3_ at pH 4.0. To prevent microbial growth, NaN_3_ was added (Orgell, 1955). Four CM discs per fruit were collected from the neck and from the body in the region of maximum fruit diameter. A total of 30 ‘Joly’ fruit from each shape category was used.

After isolation, CMs were rinsed three times with deionized water. Adhering cellular debris was removed by ultrasonication at 35 kHz for 10 min (RK 510; Bandelin electronic, Berlin, Germany). All achenes were removed manually from the CM discs. The CMs were thoroughly dried above silica gel for 48 h and then weighed. The number of replicates was 10 with five CM discs per replicate.

### Microscopic analysis

Epidermal cells in the proximal, mid and distal regions of the neck were observed at ×300 using a digital microscope (VHX-7000; Keyence, Osaka, Japan). Calibrated images were taken from normal-shaped (7.4 ± 0.4 g) and necked (7.9 ± 0.4 g) ‘Clery’ fruit of approximately the same mass. The number of fruits per category was 10. The cell width and length, and the angle between the longitudinal axis of the cell and that of the fruit were quantified by image analysis. The number of replicates was 50.

Microcracks were studied using the procedure by Peschel & Knoche (2005). Briefly, normal and necked fruit were incubated in 0.1% acridine orange (Carl Roth, Karlsruhe, Germany) for 5 min and rinsed with deionized water. The proximal, mid and distal regions of the neck surface were inspected using a binocular microscope (Leica MZ10F; Leica Microsystems GmbH, Wetzlar, Germany) equipped with a GFP plus filter (480–440 nm excitation wavelength, ≥510 nm emission). Calibrated micrographs were taken. The orientations of the microcracks relative to the longitudinal axis of the fruit was measured on necked fruit. There were no (or only very few) microcracks on normal-shaped fruit. The total number of replications per region was 40.

### Cracking induction

The tissue tension in different regions of the fruit surface was evaluated using gaping assays (Skene, 1980; Grimm et al., 2012). Following incisions of the fruit surface, the resulting wound gapes as the tissue strain is gradually released. In a mature strawberry fruit, the flesh is under compression, whereas the skin is under tension. As a result, an incision in the fruit surface gradually gapes and the extent of gaping is a measure of the tissue tension released. The following experiments were conducted: First, a time course of gaping was determined on the distal portion of necked fruit by making a latitudinal or a longitudinal standard cut of 5 mm length and 1 mm depth using a razor blade.

Calibrated images of the resulting wound were taken using a binocular (MZ6 microscope; Leica Mikrosysteme GmbH, Bensheim, Germany) equipped with a video camera (Hitachi Denshi Europa GmbH, Rodgau, Germany) 1 min and 4, 8, 24 and 48 h after making the incision. Fruit were held at 100% RH to minimize transpiration. Gape width was measured by image analysis. Second, tissue tension was investigated in different regions of the fruit surface of normal and necked fruit. Latitudinal and longitudinal standard cuts were made in the proximal, mid and distal regions of the neck and in the region of maximum diameter of the body. Fruit were then incubated in deionized water for 4 h. Calibrated images were taken, and the gape widths and gape lengths measured. The number of replicates per treatment was 10.

Cracking was induced by incubation in deionized water. In the first experiment, a time course of cracking was established by incubating necked fruit and counting the number of fruit that had cracked in the longitudinal and latitudinal directions in the neck region after 0, 2, 4, 6 and 24 h. The total number of fruits used was 50. In the second experiment, cracking was compared between normal and necked fruit. Both categories of fruit were incubated in deionized water to induce cracking. The directions and positions of cracks were recorded after 4 h of incubation. The number of replicates was 40.

### Terminology

Although in botanical terms, the ‘fruit’ of the strawberry is a pseudocarp; achenes are the real fruit containing the seeds and the ‘calyx’ is a calyculus formed by sepals in the inner ring and bracts or epi-sepals in the outer ring (Darrow, 1966; Hollender et al., 2012), for conventional terms in this manuscript we will refer to these strawberry parts as the ‘fruit’ and ‘seeds’ and ‘calyx’, respectively.

### Data analyses

Results from counting and ratings were subjected to Chi-square tests and the main effects were determined by analysis of deviance (like ANOVA-tables) on generalized linear models (Pekár & Brabec, 2016). Continuous data were analyzed by analysis of variance (ANOVA). Means were compared using Tukey’s test at *p* = 0.05. All statistical analyses were performed using R (version 3.5.1; R Core Team, 2018). Unless frequencies of counting are shown, data are presented as means ± standard errors.

## Results

Most cracking occurred in the necked fruit (those with pronounced necks) as compared to normal-shaped fruit (Fig. 1D, Table 1). Macrocracks were observed mainly in the seedless zone of the necked fruit (Figs. 1D–1F). Cross-sections of normal-shaped and necked fruit indicate the absence of seeds in the neck region. Vascular bundles in the neck were parallel to the longitudinal axis of the fruit. The fruit body region was characterized by the presence of seeds and of vascular bundles which were branched and connected radially with the seeds on the fruit surface (Figs. 1B and 1C).

**Table 1:** Frequency of cracking on small, medium size and large normal-shaped or necked strawberry cultivars.

Cultivar	Shape	Frequency of fruit (%)	Frequency of cracked fruit (%)			
			Small^c^	Medium	Large	Mean
Flair	Normal	57.3	5.0	22.1	45.0	24.0
	Necked	42.7	11.1	43.3	64.0	39.5
Lambada	Normal	34.7	10.0	10.7	6.7	9.1
	Necked	65.3	13.2	24.6	48.9	28.9
Joly	Normal	10.0	11.7	20.0	10.0	13.9
	Necked	90.0	69.1	74.0	83.3	75.5
Dream	Normal	30.0	32.7	45.0	75.0	50.9
	Necked	70.0	79.0	86.9	95.6	87.2
Overall mean	Normal	33.0a^b^	14.8	24.5	34.2	24.5a^a^
	Necked	67.0b	43.1	57.2	72.9	57.7b
	Mean	50.0	29.0a^a^	40.8b	53.5c	41.1

**Notes:**

a Means followed by the same letter are not significantly different according to Tukey’s studentized range test at *p* = 0.05.

b Means followed by the same letter are not significantly different according to the Chi-square-test at *p* = 0.05.

c Small fruit <11 g, medium 11–22 g, large >22 g.

Field ratings of fruit of the different cultivars confirmed that the frequency of cracking was consistently greater in necked fruit as compared to normal-shaped fruit. Also, large fruit was clearly more susceptible to cracking than medium-sized or small fruit (Table 1). There was no significant interaction between fruit size and cultivar.

**Figure 2:**
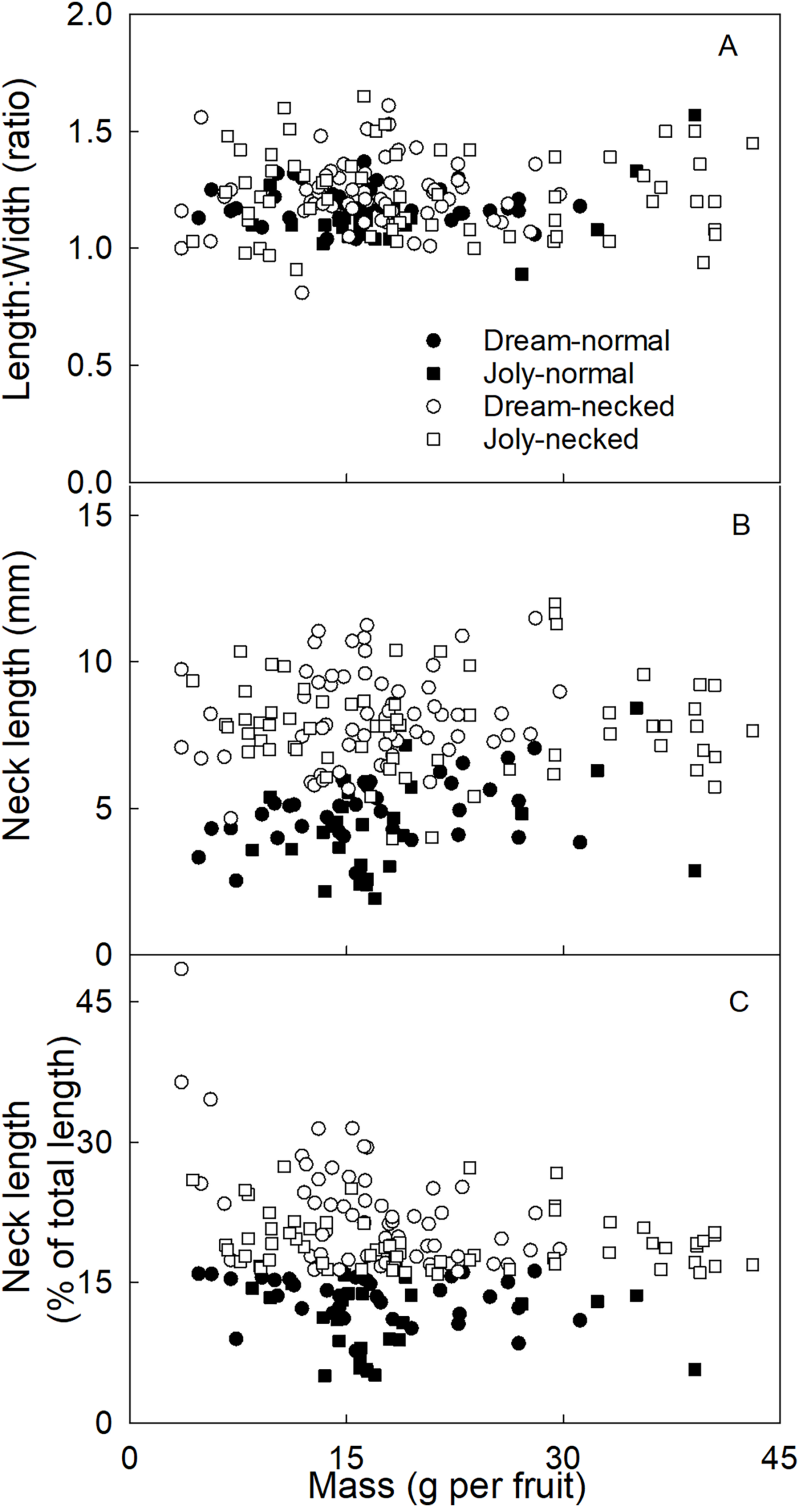
Characterizing strawberries without and with neck. Effect of fruit size on (A) the ratio between maximum length and width of the fruit, (B) the absolute and (C) relative length of the neck.

To distinguish necked from normal-shaped fruit in subsequent analyses, objective criteria for characterizing both categories of shape were determined. There was no consistent difference in the length/width ratio between normal and necked fruit (Fig. 2A). However, necked fruit had longer necks than normal fruit (Fig. 2B). This distinction was consistent for small fruit but less so for larger fruit. We determined that the most useful criterion for differentiating between necked and normal-shaped fruit was to determine the relative (%) length of the neck, *i.e*., neck length/total fruit length ×100. Fruit with necks longer than 16% of total fruit length were classified as ‘necked’. Fruit with relative lengths below 16% were categorized as ‘normal-shaped’ (Fig. 2C).

**Figure 3:**
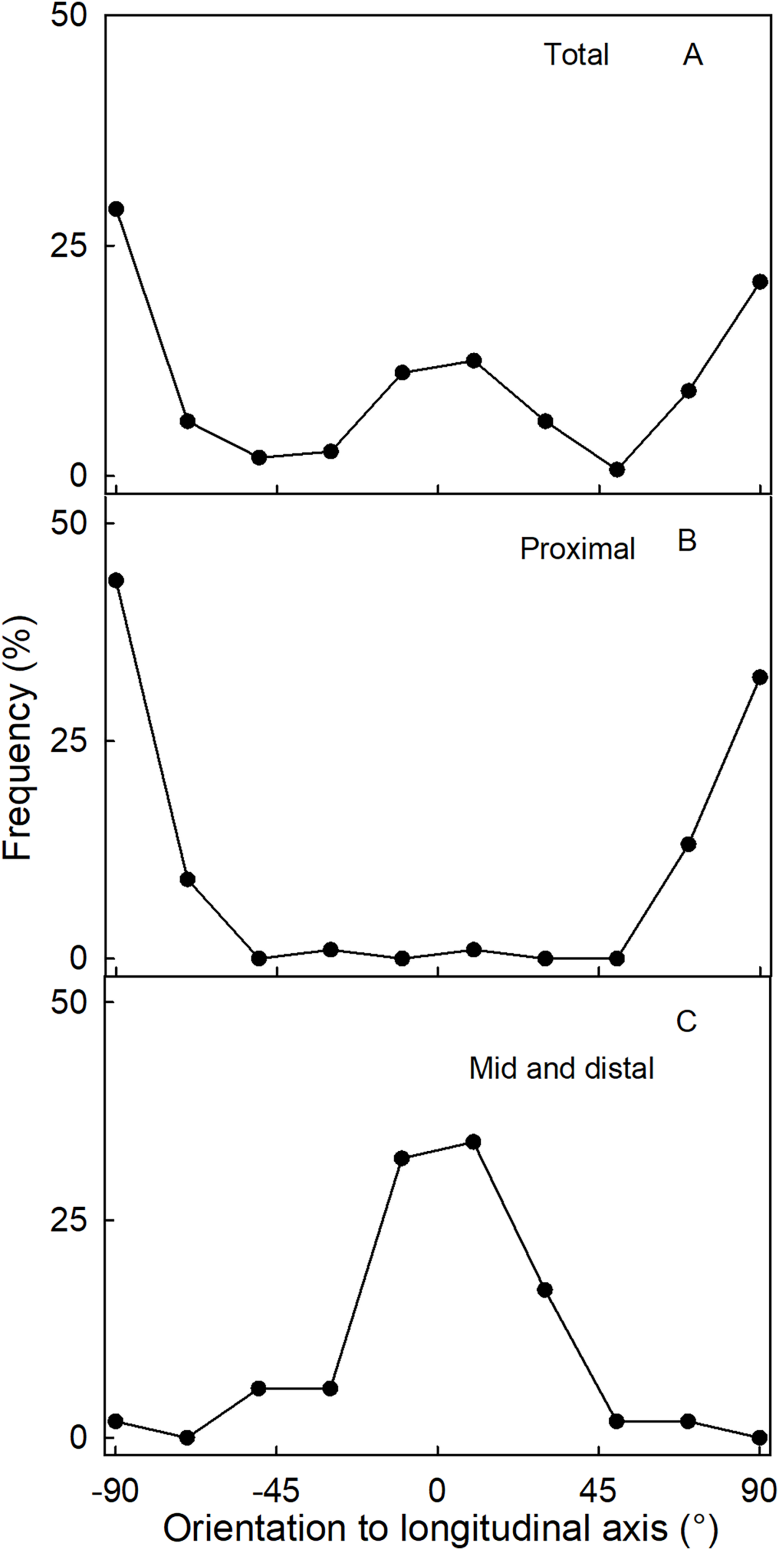
Frequency distribution of the orientation of macrocracks on a strawberry fruit with neck. (A) All macrocracks of the neck. (B) Macrocracks of the proximal region of the neck. (C) Macrocracks in the mid and distal regions of the neck of ‘Dream and Joly’ strawberries. The macrocrack orientation was measured relative to the fruit’s longitudinal axis.

The orientation of macrocracks in the neck was predominantly in the latitudinal direction (at ±90° to the fruit’s long axis) and the longitudinal direction (at 0°) (Figs. 1E, 1F and 3A). In the proximal portion of the neck, near the calyx-receptacle junction, the macrocracks were most frequently latitudinal in orientation (Figs. 1E, 1F and 3B). In contrast, in the mid and distal regions of the neck, the macrocracks were mainly longitudinal (Figs. 1E and 3C).

Cuticle thickness, as indexed by cuticular mass per unit area, was greater in the neck region than in the body for both normal-shaped and necked fruit. In addition, the cuticle of the fruit body was thicker in necked fruit than in normal-shaped fruit (Table 2).

**Table 2:** Mass of the cuticular membrane (CM) per unit area in the neck and the body regions of normal-shaped or necked ‘Joly’ strawberries.

	CM (g m^−2^)		
Fruit zone	Normal	Necked	Mean
Neck	0.88 ± 0.06a^a^	0.88 ± 0.05a	0.88 ± 0.03
Body	0.42 ± 0.02c	0.55 ± 0.02b	0.48 ± 0.02
Mean	0.65 ± 0.02	0.71 ± 0.02	0.68 ± 0.02

**Note:**

a ANOVA results indicated a significant interaction between main factors. Means followed by the same letter are not significantly different according to Tukey’s studentized range test, *p* = 0.05.

**Figure 4:**
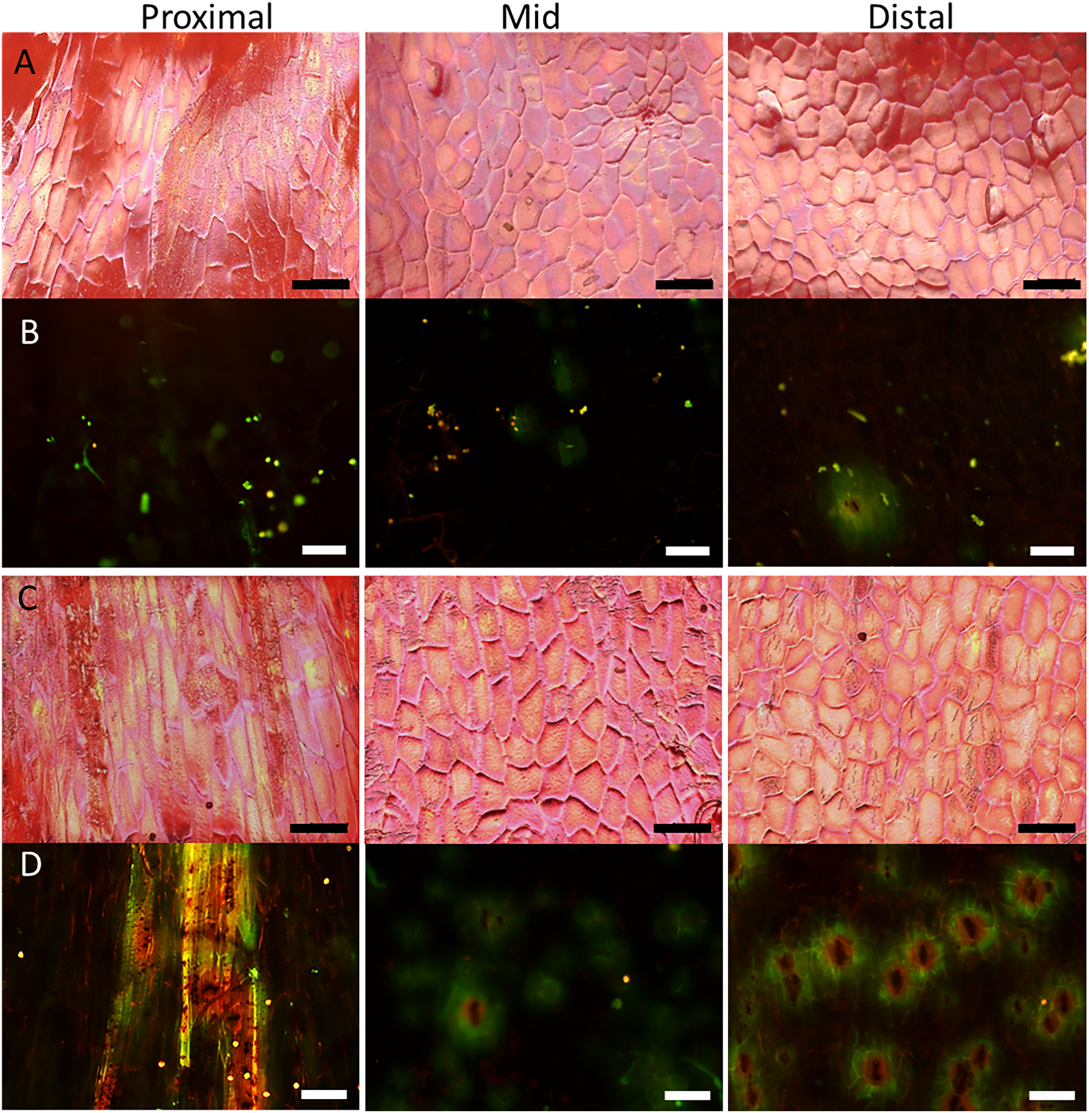
Micrographs of strawberry fruit without and with neck. Surface scans taken on a digital microscope (A, C) and fluorescence micrographs taken on a fluorescence binocular (B, D) of the seedless neck of (A, B) normal-shaped and (C, D) necked ‘Clery’ strawberries. Left column: Proximal neck. Centre column: Mid neck region. Right column: Distal neck region. Bars in A, C = 150 µm; B, D = 200 µm.

**Figure 5:**
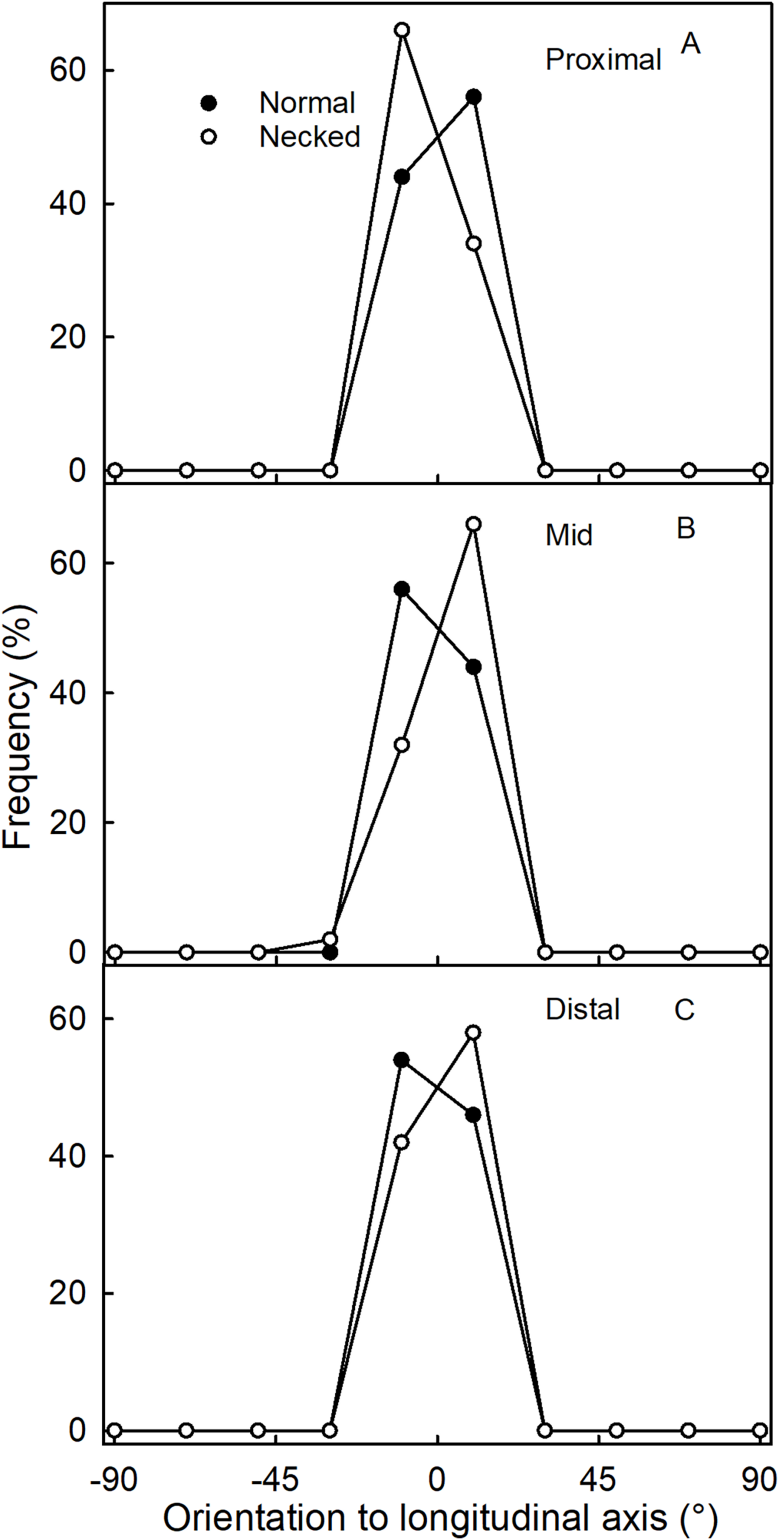
Frequency distribution of the orientations of epidermal cells on a strawberry fruit. (A) Proximal neck. (B) Mid neck. (C) Distal neck. The cell orientations were measured relative to the fruit’s longitudinal axis of normal-shaped and necked ‘Clery’ strawberries.

Surface scans of the neck region revealed that epidermal cells were polygonal in shape (Figs. 4A and 4C). In both normal-shaped and necked fruit, the longer axes of the epidermal cells were consistently parallel to the longitudinal axis of the fruit. This is indicative of skin stretching along the longitudinal fruit axis (Fig. 5). The epidermal cells of necked fruit were markedly more stretched longitudinally than those of normal-shaped fruit, particularly in the proximal and mid regions of the neck as indexed by higher aspect ratios (epidermal cell length/width) (Figs. 4A and 4C; Table 3).

**Table 3:** Ratio of length to width (‘aspect ratio’) of epidermal cells in the proximal, mid and distal regions of necks of normal-shaped and necked ‘Clery’ strawberries.

Zone	Aspect ratio (length: width)		
	Normal	Necked	Mean
Proximal	3.3 ± 0.1b	4.6 ± 0.2a^a^	4.0 ± 0.1
Mid	2.7 ± 0.1d	3.2 ± 0.2c	2.9 ± 0.1
Distal	2.0 ± 0.1e	2.1 ± 0.1e	2.1 ± 0.1
Mean	2.7 ± 0.1	3.3 ± 0.1	3.0 ± 0.1

**Note:**

a ANOVA results indicated a significant interaction between main factors. Means followed by the same letter are not significantly different according to Tukey’s studentized range test, *p* = 0.05.

**Figure 6:**
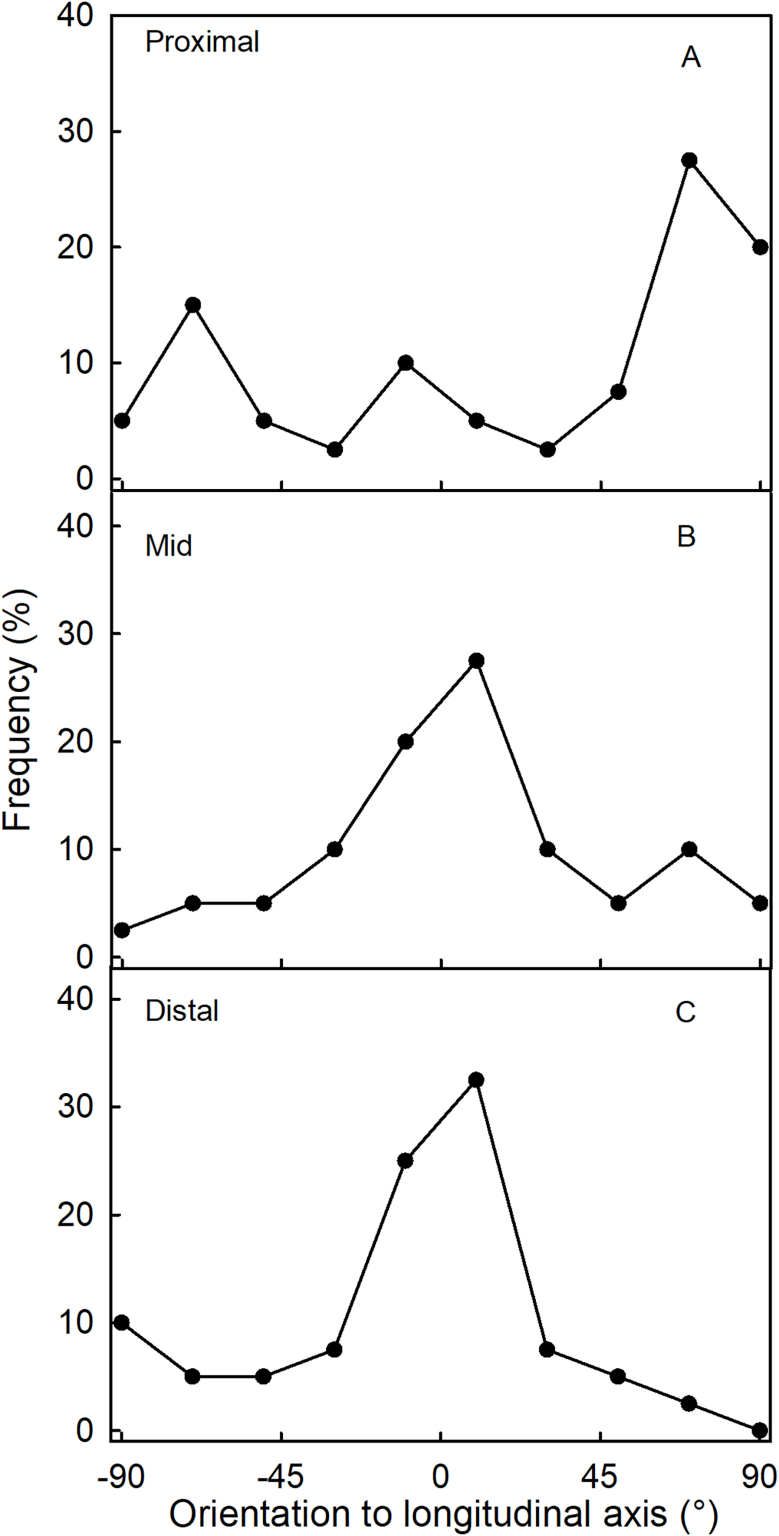
Frequency distribution of the orientations of microcracks on a strawberry fruit with neck. (A) Proximal neck. (B) Mid neck. (C) Distal neck. The orientations of microcracks were measured relative to the fruit’s longitudinal axis of necked ‘Clery’ strawberries.

Fluorescence microscopy indicates a large number of microcracks in the proximal, mid and distal regions of the necks of necked fruit as compared to the less pronounced necks of normal-shaped fruit (Figs. 4B and 4D). Microcrack orientation in the proximal region was mainly latitudinal (Fig. 6A) while that in the mid and distal regions was mainly longitudinal (Figs. 6B and 6C). Due to the lack of microcracks in normal-shaped fruit, this analysis could only be performed in the necked fruit.

**Figure 7:**
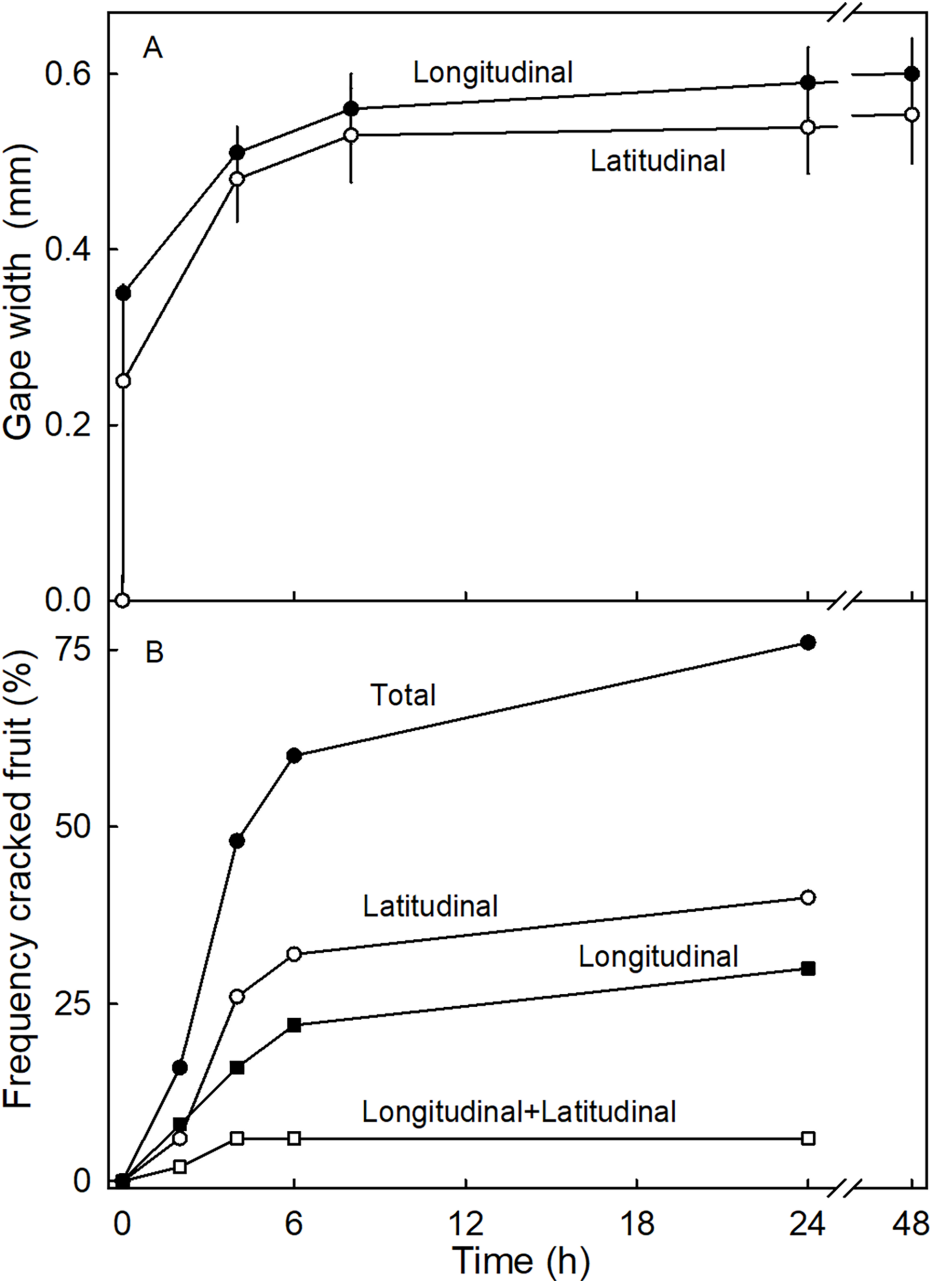
Time courses of strain release and crack formation in strawberry fruit. (A) Strain release following incisions of the fruit surface with longitudinal or latitudinal orientations. The strain release was indexed by measuring the gape size of the cut. (B) Formation of macrocracks of ‘Joly’ strawberry fruit incubated in deionized water. Fruits that cracked longitudinally *or* latitudinally, or longitudinally *and* latitudinally were counted separately.

The time course of the gaping test revealed a rapid increase in gape width that slowed with time as gape width approached an asymptote. There was little difference in gaping behavior between incisions in the longitudinal and latitudinal directions (Fig. 7A). Comparing gaping in different regions between fruit without and with necks, indicated significantly higher strain relaxation in necked fruit than in normal-shaped fruit (Table 4). Markedly more gaping was observed after the incision when fruit were incubated in water than when held at 100% RH. Latitudinal incisions resulted in less gaping in the proximal region of the neck than in the mid region (Table 4). No significant differences were found in the gape length between normal and necked fruit (Data S1).

**Table 4:** Gaping following artificial incisions and frequency of cracking of strawberries.

Crack or cut direction	Zone		Gape width (mm)			Frequency of cracks (%)	
			Normal	Necked	Mean	Normal	Necked
Latitudinal	Neck	Proximal	0.7 ± 0.1	0.9 ± 0.1	0.8 ± 0.1a^a^	3.4	25.4
Latitudinal	Neck	Center	1.2 ± 0.1	1.2 ± 0.1	1.2 ± 0.1bc	1.7	1.7
Latitudinal	Neck	Distal	1.0 ± 0.1	1.2 ± 0.1	1.1 ± 0.1abc	3.4	3.4
Latitudinal	Body	Max. diameter	0.8 ± 0.1	1.1 ± 0.1	1.0 ± 0.1ab	1.7	3.4
Longitudinal	Neck	Distal	1.1 ± 0.1	1.5 ± 0.1	1.3 ± 0.1c	8.5	25.4
Longitudinal	Body	Max. diameter	0.9 ± 0.1	1.2 ± 0.1	1.0 ± 0.1abc	5.1	11.9
Other	Body	–	–	–	–	1.7	3.4
Total/Mean			0.9 ± 0.1a	1.2 ± 0.1b	1.1 ± 0.1	25.4a^b^	74.6b

**Notes:**

a Means followed by the same letter are not significantly different according to Tukey’s studentized range test, *p* = 0.05.

b Means followed by the same letter are not significantly different according to the Chi-square-test at *p* = 0.05.

Fruit were immersed in water. Gaping and frequency of cracking were assessed in the proximal, mid and distal neck regions and in the bodies of normal-shaped and necked ‘Clery’ strawberries.

Incubating fruit in deionized water induced macrocracks in about 75% of necked fruit (Fig. 7B). Cracking was rapid, as indexed by half of the fruit cracked within about 4.4 h of starting incubation. At this time the fruit had taken up about 506 mg of water (5% of fruit mass). Necked fruit cracked with a markedly higher frequency than normal-shaped fruit (Table 4). Most of the macrocracks were oriented latitudinally in the proximal region of the neck and longitudinally in the distal region of the neck (Table 4).

## Discussion

Our main findings are: (1) growth strain is the principal driver of cracking of strawberries and (2) necked fruit are more susceptible than normal-shaped fruit and (3) water uptake greatly exacerbates cracking.

### Growth strain—the driver for cracking

A strawberry differs from a true fruit in *sensu stricta* in several respects. First, the body of the strawberry is comprised mostly of receptacle tissue and the actual fruits are the small pips (achenes) embedded in and on this receptacle. As such, the strawberry is a pseudocarp or a false fruit. Second, a strawberry fruit develops over a very short period of time compared to other soft fruits (*i.e*., 20–40 days, not 15–20 weeks). Thus, the skin of the receptacle (*i.e*., the ‘fruit’ surface) is subject to very rapid rates of strain compared to the skins of other soft fruits (Darrow, 1966; Batal, Weigle & Foley, 1970; Considine, 2015; Khanal, Grimm & Knoche, 2011; Grimm et al., 2012).

Several observations indicate the key role of growth strain in strawberry fruit cracking. (1) The percentage of cracking was depended on fruit size. The number of cracked fruit increased as fruit size increased. Interestingly, fruit with an extended neck always cracked more than normal-shaped fruit. (2) The skin ‘gaped’ following artificial incisions. Gaping indicates that the flesh is held under compression by a strained skin. (3) The extent of gaping and the distribution of macrocracks across the fruit surface were closely related. Necked fruit gaped more and cracked more than normal-shaped fruit. Furthermore, for longitudinal gapes, the width and the number of longitudinal macrocracks were largest in the zone of the maximum body diameter and the distal neck in its close vicinity, as compared to other regions or to latitudinal macrocracks. (4) There was marked elongation of cells as indexed by higher length/width aspect ratios in regions of higher incidence of cracking. Again, necked fruit that had the higher percentage of cracking also had higher aspect ratios than normal-shaped fruit. In addition, cells were the most extended in the proximal neck region which also had the highest number of macrocracks. (5) Cuticle thickness was lower in the body than in the neck, which is consistent with the larger surface area of the body *vs* the neck. As in other fruit crops, cuticle deposition in strawberries is likely to be limited to the early stages of development and any expansion occurring after cessation of cuticle deposition simply ‘dilutes’ the cuticle (*i.e*., the same amount of cuticular material becomes spread over an increasing area of skin) resulting in a steady decrease in cuticular thickness (Knoche et al., 2004; Becker & Knoche, 2012). Whether the resulting strain in the cuticle is elastic or plastic is not known.

These arguments indicate that growth strain provides a conclusive explanation for the behavior of cracking in strawberries and for the higher susceptibility of the necked *vs* the normal-shaped fruit.

### Role of water uptake in cracking

Water uptake through the fruit surface exacerbates rain-cracking in strawberry, but also in a range of other soft fruit including sweet cherry (Christensen, 1996; Knoche & Winkler, 2017) and grape (Considine, 2015). Evidence for water uptake exacerbating cracking is based on the following observations: First, surface wetness increases microcracking of the cuticle (Hurtado & Knoche, 2021). Second, water-induced macrocracks did not differ in size or orientation from ‘natural’ macrocracks that were not induced by water uptake, Third, water uptake increased growth strain in the skin, as indexed by more gaping after incubation in water as compared to holding fruit at 100% RH. Fourth, the abscission zones of petals and microcracks in the calyx-receptacle junction, around the base of stamina in the seedless neck are further openings, where the cuticle’s barrier function in controlling water transfer is impaired. In these regions, rapid uptake of water occurs as demonstrated by infiltration with the fluorescent tracer acridine orange (Hurtado & Knoche, 2023). An opening in the cuticle acts in a way analogous to an aperture in an optical instrument. It ‘focusses’ water uptake into a highly localized region of the fruit skin. This uptake occurs by viscous flow (Hurtado et al., 2021), which is much more rapid than by diffusion through an intact cuticle (Winkler et al., 2016). The close proximity of these preferential pathways to the vascular bundles in the seedless neck, ensures rapid water uptake and subsequent movement which further contributes to the greater incidence of cracking in this region.

How do microcracks subsequently extend to macrocracks? An explanation for the mechanism of extension and the role of water uptake therein may be found in the ‘Zipper’ model. This model accounts for rain cracking in sweet cherries (Winkler et al., 2016). It also explains the water soaking disorder of strawberries (Winkler et al., 2016; Hurtado & Knoche, 2021). Briefly, water uptake through a microcrack in the cuticle causes individual underlying cells to burst. Their cell contents are released into the apoplast. Sweet cherries and strawberries are rich in organic acids (70 mM malic acid in sweet cherry *vs* 23 mM malic acid and 39 mM citric acid in strawberry (Herrmann, 2001)). These organic acids increase the membrane permeability of the adjacent cells, causing further leakage, a weakening of the cell walls, and triggering a chain reaction that extends radially into the flesh and longitudinally into the skin like a “zipper” (Winkler et al., 2016). This chain reaction extends a (shallow) microcrack into a (much deeper) macrocrack or—as the busting of skin cells proceeds—leads to water-soaking (Hurtado & Knoche, 2021). The latter was also observed in our time course of cracking induction in this study.

### A simple model to explain macrocracking of strawberry

**Figure 8:**
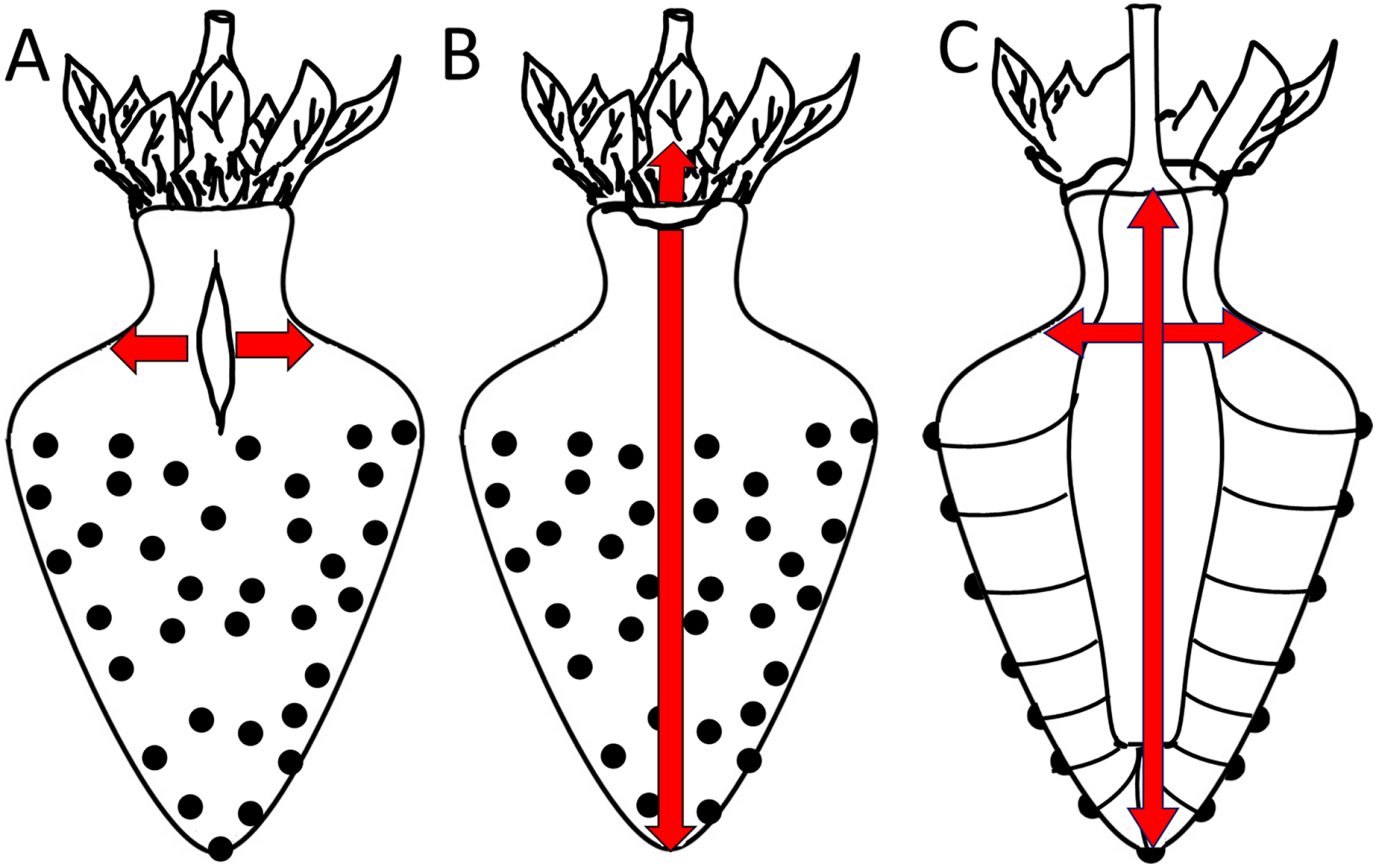
Sketch of growth strains and crack formation in strawberry fruit. (A, B) Direction of growth strains and the resulting directions of crack formation in the neck region of necked fruit. (C) Vascular bundles connecting peduncle to seeds.

In this section, we focus on macrocracks. Macrocracks arise from microcracks. Macrocracks on strawberries are highly orientated. The orientation is consistent within regions but differs between regions. Two different orientations dominate: In the fruit body and the distal neck region, macrocracks are orientated longitudinally. In contrast, in the proximal neck region latitudinally-orientated macrocracks dominate. These observations are in agreement with Herrington et al. (2011). Furthermore, the orientations of macrocracks and microcracks are identical, this implies that macrocracks arise from the extension of microcracks. Our study established that fruits with extended necks are particularly prone to both micro- and macrocracking. To explain these observations, we propose a model that relates the frequency and the orientation of micro- and macrocracks to the growth strains in the strawberry fruit (Fig. 8).

A longitudinal orientation is favored by one or several of the following factors. (1) The cone shape of a strawberry favors cracking in a longitudinal direction. (2) Most longitudinal cracks occur in the regions where the diameter of the fruit is largest and in the distal region of the neck. In the physicist’s mathematical analysis of a ‘thin-walled, cylindrical, pressure vessel’, the tangential stress in the wall (σ, N m^−2^) at a given pressure (P, N m^−2^) is directly proportional to the radius (R, m) of the vessel, divided by its wall thickness (t, m) according to the equation (Nobel, 1999):

$\sigma = \displaystyle{{P\; \times \; R} \over t}$

This physical model holds for the strawberry within the body and within the neck. However, it does not explain why the neck cracks more frequently than the body, despite the neck having a smaller diameter (Fig. 8). Based on the difference in diameter, we would expect the neck to have fewer macrocracks, not more microcracks. Two explanations may be offered. The first explanation relates to the absence of seeds (achenes) in the neck region. In a strawberry, every seed is supplied by a vascular bundle. In mechanical terms, a fruit’s vascular bundles represent ‘structural reinforcements’ (c.f., steel-reinforced concrete) embedded in the soft and extensible parenchyma of its receptacle. In the neck region (no seeds), the vascular bundles run parallel to the fruits’ long axis. In contrast, beyond the neck in the fruit’s body region (with seeds), the vascular bundles also branch off in the radial direction to supply the seeds on the fruit surface. The radial orientation of this vasculature now provides structural reinforcement in the body of the fruit also in the radial direction—this radial reinforcement is lacking in the neck (Fig. 8). In consequence, the radial reinforcement restricts cracking in the body, but does not do so in the neck, which lacks radial reinforcement. This explanation also accounts for the ‘corrugated’ nature of the strawberry fruit surface and the location of the seeds in shallow depressions. Since expansion of the parenchyma is restricted by the radial vascular bundles, the seeds are ‘pulled’ down into the expanding parenchyma. That the strawberry forms a neck may simply be the result of greater longitudinal extension in this region where fruit diameter is lower. In this region, elongation per unit cross section will be largest. This explanation would account for (1) the higher aspect ratio of epidermal cells in the neck of a necked fruit *vs* in the neck of a normal-shaped fruit and (2) the decrease in frequency of longitudinal macrocracks within the neck region from the proximal to the distal end as the diameter of the neck increases.

The second explanation relates to the corrugated nature of the strawberry surface. The seeded region of a strawberry has an enlarged surface area due to the tiny ridges and valleys that form the multiple tiny indentations of the epidermis that accommodate the seeds. In contrast, the seedless neck is flat. So, as fruit volume expands rapidly, the corrugated fruit body has a relatively large surface that can accommodate a rapid area increase by a conformational change (it can extend by flattening out), rather than by stretching *per se*. The non-corrugated, seedless neck does not have this advantage. Hence, the seedless neck lacks this mode of area increase and so cracks more easily than the seeded body of the fruit.

Latitudinal cracks were limited to the proximal neck region. The following factors are likely to be causal: (1) The junction between the calyx and the receptacle is likely to be weakened by discontinuities in the skin (corky, stiff patches) associated with the petal and the stamen abscission zones. (2) The calyx, petal and stamen bundles are all linked into the shoot vasculature to secure water supply for transpiration. However, distal to the staminal whorl, the number of vascular bundles decreases, as those that continue connect only to the seeds. Since vascular bundles offer significant structural support to any tissue in which they are embedded, the abrupt decrease in bundle numbers (and thus structural support) beyond the staminal whorl represents a relative weakening of these distal tissues, especially in terms of longitudinal extension. This discontinuity may serve as a stress concentrator that focuses longitudinal growth to the proximal neck region. (3) The proximal neck region is also an area of preferential water uptake. Water uptake exacerbates cracking.

The above arguments seem to account fully for the observed differences in cracking susceptibility between necked and normal-shaped strawberry fruit and also for the consistent patterns of crack orientation in the neck region of necked fruit.

## Conclusion

The model proposed here offers a satisfactory explanation for the failure patterns of cracking in strawberries and this pattern’s close relationship to fruit shape and structure. Our results demonstrate that fruit with extended necks are especially susceptible to cracking. The frequency of necking in strawberry depends on genotype, but also on as-yet-unknown environmental factors. Further research is needed to unravel the mechanism of neck formation. Protected cultivation is a useful way for reducing cracking in necked fruit. As, in unprotected cultivation systems, the seedless neck region is likely to suffer extended periods of surface wetness, that will increase cuticular microcracking, the microcracking then soon progresses to macrocracking, that extends these cracks deep into the flesh.

## Supplemental Information

10.7717/peerj.15402/supp-1 Supplemental Information 1 Raw data for tables and figures.

## Additional Information and Declarations

### Data Availability

The following information was supplied regarding data availability: The data used to generate tables and figures are available in the Supplemental File.
